# Effects of Feeding Regimens and Dietary Methionine Level on Growth Performance and Feather Growth of Meat Ducks from 29 to 49 Days of Age

**DOI:** 10.3390/ani15111528

**Published:** 2025-05-23

**Authors:** Weicheng Shu, Qiufeng Zeng, Shiping Bai, Jianping Wang, Xuemei Ding, Yue Xuan, Keying Zhang

**Affiliations:** Key Laboratory of Animal Disease-Resistance Nutrition Ministry of Education, Ministry of Agriculture and Rural Affairs, Key Laboratory of Sichuan Province, Animal Nutrition Institute, Sichuan Agricultural University, Chengdu 611130, China; shuwc23@gmail.com (W.S.); zqf@sicau.edu.cn (Q.Z.); shipingbai@sicau.edu.cn (S.B.); wangjianping@sicau.edu.cn (J.W.); dingxuemei0306@163.com (X.D.); 71128@sicau.edu.cn (Y.X.)

**Keywords:** feed restriction, methionine, feather growth, growth performance, duck

## Abstract

This study was conducted to evaluate the effects of feeding regimens and dietary methionine (Met) levels on the growth performance and feather growth of Cherry Valley ducks from 29 to 49 days of age. A 2 × 3 factorial arrangement was employed, consisting of two feeding regimens (ad libitum or “5+2” feed restriction: 5 days ad libitum + 2 days of fasting) and three levels of dietary Met (0.32%, 0.40%, or 0.48%). A total of 600 29-day-old male Cherry Valley ducks (initial body weight: 2.18 ± 0.12 kg) were randomly assigned to six treatment groups, with 10 replicates of 10 ducks each. Dietary Met as a main effect did not significantly influence growth performance *(p* > 0.05). The “5+2” feed restriction regimen reduced the 49-day final body weight, body weight gain, and feed intake (days 29–49) compared with ad libitum feeding, with a concurrent increase in the feed conversion ratio (*p* < 0.01). Body weight uniformity in the “5+2” feed restriction group was 4.33% higher than in the ad libitum group, though this difference was not statistically significant (*p* > 0.05). In addition, the feeding regimen tended to affect the feather weight and feather ratio, with the “5+2” regimen reducing the feather weight (*p* < 0.1) but increasing the feather ratio (*p* < 0.1) relative to ad libitum feeding. Higher dietary Met levels showed a tendency to improve the feather length and overall feather score *(p* < 0.1). A significant interaction was observed between the feeding regimen and Met level for wing feather length (*p* < 0.05). In conclusion, the “5+2” feed restriction impaired the growth performance and absolute feather yield in meat ducks. However, dietary Met levels interacted with feeding strategies to affect feather development. Specifically, under ad libitum feeding conditions, moderate-to-high Met supplementation (0.48% Met) appeared to be beneficial for feather yield, whereas lower Met levels (0.32% Met) were more effective for enhancing feather mass under restricted feeding. These findings highlight the potential to tailor Met supplementation according to feeding regimen to optimize both production and feather quality in ducks.

## Simple Summary

This study investigated the effects of different feeding regimens (ad libitum versus “5+2” restriction: 5-day ad libitum + 2 days of fasting) and varying dietary methionine (Met) levels (0.32%, 0.40%, and 0.48%) on growth performance and feather characteristics in Cherry Valley ducks aged 29–49 days. Feed restrictions significantly reduced the final body weight, weight gain, and feed intake and impaired feed conversion (*p* < 0.05), yet it improved body weight uniformity by 4.33% (*p* > 0.05). While dietary Met levels did not significantly affect overall growth performance, an interaction with the feeding regimen was observed for the feather mass, as 0.48% Met maximized the feather mass under ad libitum feeding, whereas 0.32% Met was optimal for ducks under the “5+2” restriction. Furthermore, higher dietary Met levels tended to improve feather length and quality scores (*p* < 0.1). These findings offer guidance for duck producers seeking to balance meat yield with feather quality through tailored feeding strategies and appropriate Met supplementation.

## 1. Introduction

Meat ducks are a key agricultural resource in China, prized for both their meat and feathers. Reflecting wider consumer trends, there is growing demand for slow-growing poultry, driven by an increasing focus on meat quality [1]. Consequently, various feed restriction strategies are gaining traction in Chinese duck production. Among these, the “5+2” intermittent fasting regimen—five days of ad libitum feeding and two days of fasting—draws from practices effectively used in breeding chicken management to mitigate the potential negative effects of prolonged fasting [2] and is being explored in duck production to control the body weight at about 2.5 kg at 50 days of age. Feathers play a vital role for poultry, providing critical thermal insulation that can reduce maintenance energy requirements and thereby improve feed conversion efficiency [3,4]. Moreover, feathers are an economically important by-product in duck production [5]. But feather defects such as feather pecking, abnormal molting, and insufficient feather coverage are frequently observed, and they can significantly undermine slaughter quality and reduce net profitability [6,7,8]. Current findings regarding the effects of feed restriction on feather growth remain inconsistent. For instance, Arrazola [9] reported improved feather coverage under a “4+3” feed restriction regimen combined with dietary dilution, compared with ad libitum feeding. Similarly, Morrissey [10] demonstrated that alternate-day feeding enhanced feather growth relative to ad libitum feeding and found that diet dilution supplemented with appetite suppressants effectively increased satiety and improved feather integrity. Conversely, Tahamtani [11] observed detrimental effects from qualitative feed restriction on the feather condition in rooster chicks. These disparities underscore that feather development outcomes under feed restriction are critically dependent on the specific methodological approaches employed, such as fasting intervals, nutrient dilution strategies, and the overall intensity of the restriction.

Feathers are predominantly composed of keratin [12], a protein reliant on the amino acid cysteine for its synthesis [13]. Methionine (Met) is metabolically converted to cysteine via the transsulfuration pathway, thus playing an essential role in keratin synthesis and, consequently, in overall feather development [14]. As the first limiting amino acid in corn-soybean meal diets for poultry [15,16], it is recognized as a principal nutritional factor influencing feather growth for ensuring optimal feather quality [17]. For instance, studies by Chen et al. [18] found that in ovo Met administration enhances feather follicle development and feather growth. Furthermore, Guo et al. [19] identified 0.48% dietary Met as optimal for feather growth in Peking ducks during their early developmental phase (1–21 days of age). These findings collectively underscore that formulating duck diets with appropriate Met levels is crucial for achieving both satisfactory overall growth and high-quality feathering. Despite this established role, the potential for dietary Met supplementation to counteract feather growth impairments induced by feed restriction remains an underexplored area within poultry nutrition.

We hypothesized that strategically increasing the dietary Met level could improve feather development and yield, particularly in ducks subjected to feed-restricted regimens. Therefore, the objective of this study was to study the effects of dietary Met on the feather growth and quality parameters of meat ducks under ad libitum feeding or “5+2” intermittent fasting regimen.

## 2. Materials and Methods

This study was approved by the Animal Care and Use Committee of Sichuan Agricultural University (Ethic Approval Code: SICAUAC 202110-2; Chengdu, China).

### 2.1. Birds, Experimental Design, Diets, and Management

This study employed a 2 × 3 factorial design with two feeding regimens (AD: ad libitum; FR: “5+2” restricted feeding) and three dietary Met levels of 0.32%, 0.40%, and 0.48%. There were 6 treatments with 10 replicates of 10 ducks.

Initially, 720 one-day-old male Cherry Valley ducklings were obtained from Sichuan Mianying Breeding Duck Co., Ltd. (Mianyang, China). All ducklings from day 1 to day 14 received a common starter diet with 11.91 MJ/kg of apparent metabolizable energy [AME]; 19.5% of crude protein [CP]; and 0.49% Met. Then, from day 15 to day 28, the ducks were fed a diet with 0.40% Met. On day 29, 600 ducks were weighed and randomly assigned to six treatments according to their body weights.

The experimental diets were formulated based on the Chinese Feeding Standard for Ducks (NY/T 2122-2012) [20] with 0.32%, 0.40%, and 0.48% digestible methionine (Dis Met), which represented the Met recommendation of 80%, 100%, and 120%, respectively. All diets maintained identical nutrient profiles, except for their Met concentrations. The detailed ingredient compositions and calculated nutritional matrix for these diets are presented in Table 1.

The experimental trial lasted from 29 to 49 days of age. All ducks were housed in pens with dimensions of 1.00 m (length) × 0.80 m (width) × 0.60 m (height). The house was environmentally controlled for its temperature and humidity. All ducks had ad libitum access to water.

### 2.2. Data Collection

From 29 to 49 days of age, the key performance indicators were assessed. On trial days 35, 42, and 49, all ducks were individually weighed following a 12-h feed withdrawal period. The feed intake (FI) for each pen was meticulously calculated by recording the daily feed provided and feed refusals, which were measured daily. The body weight (BW), body weight gain (BWG), FI, and feed conversation ratio (FCR) were calculated. Any birds which died during the trial were weighed and used to correct the calculation of the FCR. On day 49, each duck was weighed to calculate the body weight uniformity (BWU) as follows:

$$BWU\left(\%\right)=(\frac{\;Number\;of\;duck\;within\;90\%–110\%\;of\;the\;average\;BW}{Total\;ducks\;weight})\times 100$$

### 2.3. Feather Measurements

On day 49, a total of 60 birds (1 randomly selected from each replicate) were chosen for feather assessment, including the feather lengths from various body regions (head, neck, back, chest, belly, and wings), feather score of the back, and total feather weight. Feather length was determined by trained personnel using Vernier calipers. The feather scoring criteria were adapted from the method described by Gustafson (Table 2) [21], which was performed by a trained person. Then, all of the selected birds were weighed and euthanized by exsanguination following stunning. Feathers were collected, washed, and dried in a constant-temperature oven at 65 °C. The feather weights were recorded, and the relative feather weights (feather ratios) were calculated as follows:

$$Relative\;feather\;weight(\%)=\left(\frac{Total\;feather\;weight}{live\;BW}\right)\times 100\%$$

### 2.4. Statistical Analyses

All data were analyzed using SAS statistical software (version 9.2, SAS Institute Inc., Cary, NC, USA, 2004). A two-way analysis of variance (ANOVA) was conducted, and Duncan’s multiple range test was used to determine significant differences among treatments at *p* < 0.05. A trend toward significance was defined as 0.05 ≤ *p* < 0.10. Data are presented as the mean and standard error of the mean.

## 3. Results

### 3.1. Growth Performance

As presented in Table 3, even the dietary Met intake increased with the increase in dietary Met. The dietary Met level did not influence the growth performance significantly (*p* > 0.05), with no significant interaction with the feeding regimens. Compared with AD, FR decreased the BW49, BWG, and FI significantly, while it increased the FCR (*p* < 0.01). As expected, the total Met intake increased significantly (*p* < 0.01) with rising dietary Met concentrations. Although FR improved the BWU by 4.33% compared with AD, it was not statistically significant (*p* > 0.05).

**Figure 1:**
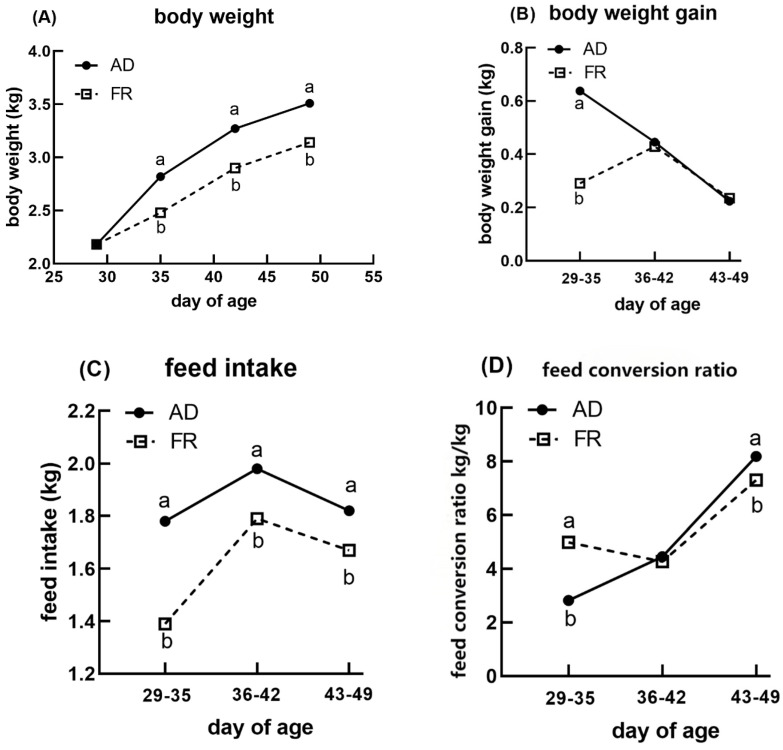
Effects of feeding regimens on the growth performance of meat ducks (29−35, 36−42, and 43−49 days of age): (**A**) body weight; (**B**) body weight gain; (**C**) feed intake; and (**D**) feed conversion ratio. ^a,b^ Means within the same days of age with different superscripts are significantly different (*p* < 0.05).

Figure 1 illustrates the duck growth performance per week. Although the BW of the ducks in the FR group at days 35, 42, and 49 was significantly lower than that of the AD group (*p* < 0.05), the most pronounced difference in BWG between AD and FR was observed at days 29–35. Later on, the BWG at days 35–42 and days 43–49 were not significantly different between FR and AD (*p* > 0.05). FR decreased the FI significantly at each stage (days 29–35, 36–42, and 43–49) (*p* < 0.05), while AD increased the FCR from day 29 to day 35, but it decreased the FCR from day 43 to day 49.

### 3.2. Feather Growth

**Figure 2:**
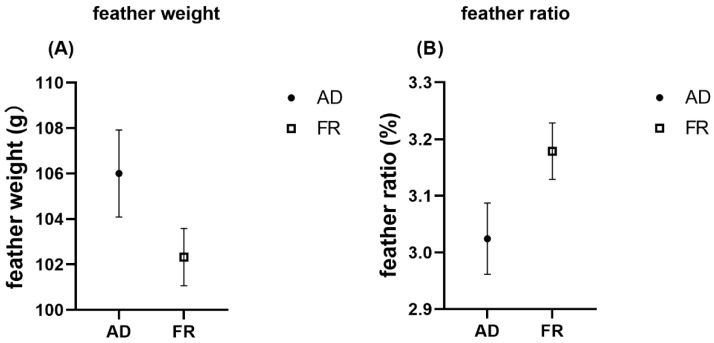
Effects of feeding regimens on feather weight and feather ratio in meat ducks. (**A**) feather weight (g); (**B**) feather ratio.

**Figure 3:**
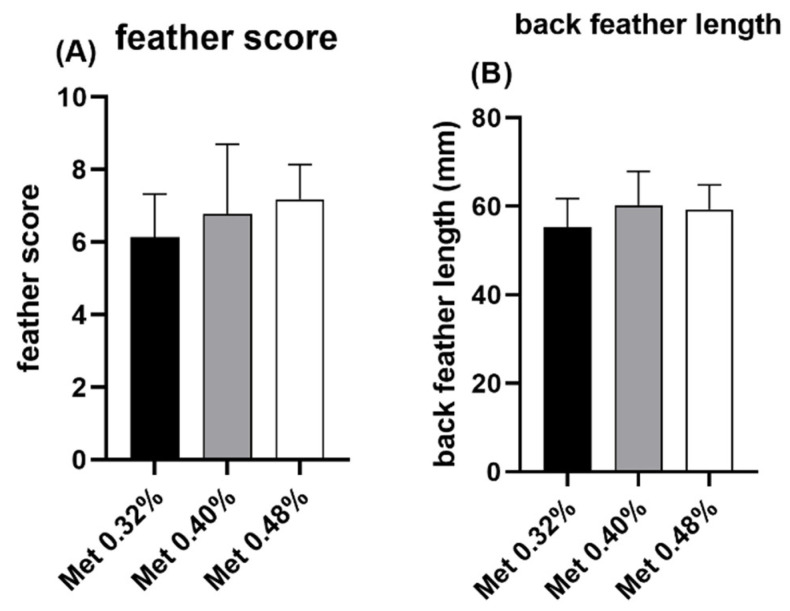
Effects of dietary methionine on feather scores and back feather length in meat ducks. (**A**) feather score; (**B**) back feather length.

The effects of feeding regimens and dietary Met on the feather weight and back feather scores in meat ducks at 49 days of age are shown in Table 4. FR tended to decrease the feather weight (*p* = 0.095, Figure 2A) while increasing the feather ratio (*p* = 0.057, Figure 2B). Under ad libitum (AD) feeding, the feather weight increased as dietary Met levels increased, but this was not the same under FR (*p* < 0.05). Back feather scores also tended to increase with the dietary Met levels (*p* = 0.075, Figure 3A).

Table 5 summarizes the effects of feeding regimens and dietary Met on the feather length at day 49. The feather lengths of the head and chest increased with the increase in dietary Met under both AD and FR, and a similar upward trend was observed for the back feather length (*p* < 0.10). In contrast, the neck feather lengths decreased as the dietary Met level increased (*p* < 0.05). FR significantly increased the chest feather length compared with AD (*p* < 0.05). A significant interaction between the Met level and feeding regimen was noted for wing feather length (*p* < 0.05), with a trend observed for belly feather length and neck feather length (*p* < 0.10). The feather length of the wing increased with the dietary Met under AD, but this was not the same under FR.

## 4. Discussion

Conventional research paradigms have predominantly employed ad libitum feeding regimens. An appropriate Met level can enhance duck meat performance, including improving body weight gain and feed efficiency [22]. However, excessively high Met supplementation can lead to toxicity and result in negative effects on growth [15]. In the present study, dietary Met at levels of 0.32%, 0.40%, and 0.48% did not significantly influence production performance under either the AD or FR regimen. This observation aligns with the results of Zhang et al. [23], who reported that supplementing DL-Met or L-Met did not influence duck growth performance during the growth phase (15–35 days). These outcomes suggest that the Met levels used in this study were adequate for meeting the growth requirements of broiler ducks without causing any negative effects.

In the present study, the “5+2” FR regimen reduced the BW, BWG, and FI but increased the FCR, resulting in growth retardation. This confirmed the findings of De Beer and Coon [24],who observed that pullet chicks fed every day were heavier than those subjected to “5+2” FR, despite having the same total feed intake. One possible explanation is that during the fasting period, the nutrients deposited during feeding may be remetabolized under the “5+2” regimen, thereby lowering efficiency. Interestingly, in our study, the ducks under “5+2” FR exhibited higher BWG and lower FRC from days 43 to 49 than the ducks fed ad libitum, suggesting that compensatory growth may have occurred. Bentley et al. [25] reported no significant difference in the body weights of Pekin ducks when ad libitum feeding was resumed following an earlier period of feed restriction. It is evident that the impact of FR on growth performance depends on the specific regimen, its intensity and duration, and the birds’ ages [26]. The findings demonstrate that “5+2” FR during days 36–49 may enable ducks to achieve body weight gain comparable to ad libitum feeding, while simultaneously lowering production costs through a reduced FI. Our results show that “5+2” FR increased the BWU by 4.33%, although this difference was not statistically significant. This is similar to the observations of Bennett and Leeson [27]. They proposed that intermittent feeding schedules, by potentially reducing competition and allowing for more equitable feed access among birds when feed is available, can foster greater flock consistency.

The addition of Met is recognized for its capacity to enhance both overall feather production and the sulfur content of feathers [13]. In the present study, under both the AD and FR feeding conditions, dietary Met at a level of 0.48% tended to improve feather scores and back feather lengths. This is similar to the observation of Zeng et al. [17], who recommended an optimal Met level of 0.48% for duck feather coverage from 15 to 35 days. Guo et al. [19] reported that the Met requirements for the maximum feather weight, 1000 down weight, and down length were 0.48%, 0.48%, and 0.49%, respectively, from 1 to 21 days. A notable finding in our study was the differential response of the feather length in various body regions to dietary Met levels. This regional specificity may reflect the inherent variations in local feather growth patterns, developmental rates, and potentially distinct Met requirements across different feather tracts. The concept of asynchronous feather development across the body was strongly supported by Xie et al. [28] in yellow-feathered broilers, who demonstrated that chest feathers emerge and mature earlier than belly feathers, illustrating such regional developmental timelines.

Interestingly, our results revealed an interaction between dietary Met and feeding regimen regarding the feather weight at day 49. Under AD feeding, the feather weight increased with higher Met levels, whereas under “5+2” FR, the opposite trend was observed. This interaction strongly suggests that the optimal dietary Met concentration for maximizing feather weight differs substantially depending on whether ducks are fed AD or FR. Previous studies have shown a quadratic increase in feather weight with Met supplementation during both embryonic development [18] and the growth phase [17], indicating that feather growth can be compromised by either insufficient or excessive Met. In the current experiment, the suitable Met level for maximizing the feather weight appeared to be lower under the “5+2” FR condition. This might be partly attributable to metabolic adaptations during the fasting periods. Nutrient deprivation can increase body protein catabolism, potentially enlarging the endogenous pool of free amino acids (including sulfur amino acids from tissue breakdown) and thereby altering the apparent dietary Met needs for feather synthesis [29]. Furthermore, we speculate that the overall rate of body weight gain significantly influences Met requirements. Given the reduced growth performance typically associated with FR, the physiological demand for Met for tissue accretion would logically decrease. Under such circumstances, higher levels of Met supplementation might exceed the ducks’ actual needs, offering no additional benefit for feather production and potentially risking adverse effects from amino acid imbalance or excess. This is something that needs to be explored further.

Furthermore, the “5+2” FR regimen elevated the feather ratio (*p* < 0.1) and feather score *(p* = 0.4), consistent with the research by Arrazola et al. [9] and Morrissey et al. [10], who found that “4+3” or skip-a-day feeding schedules improved feather scores compared with daily feeding, and they also reduced feather pecking behavior. However, the absolute feather weight was reduced under FR, likely due to the lower total nutrient intake.

## 5. Conclusions

Given the dual importance of meat ducks for both meat and feather production, particularly in contexts exploring alternative management strategies like feed restriction to optimize growth and resource utilization, this study provides critical insights into the interplay between feeding regimens and dietary methionine (Met) supplementation. The findings conclude that while a “5+2” intermittent feed restriction regimen generally impaired the overall growth performance and absolute feather yield compared with ad libitum feeding, it did not significantly alter body weight uniformity. Importantly, dietary Met levels did not show a main effect on growth performance, but a significant interaction between the feeding regimen and Met level was crucial for feather development. Specifically, under ad libitum conditions, higher Met supplementation (0.48%) appeared to be beneficial for the feather yield, whereas under feed restriction, lower Met levels (0.32%) were more effective for enhancing the feather mass. This highlights a key production significance; producers can tailor Met supplementation based on their chosen feeding strategy to more efficiently balance growth targets with the desired feather quality, potentially leading to more economical and targeted nutritional programs for meat ducks.

## Figures and Tables

**Table 1:** Composition and nutrient levels of the basal diets (dry matter basis).

Item	0.32%	0.40%	0.48%
Ingredients (%)			
Maize	53.26	53.26	53.26
Soya oil	0.50	0.50	0.50
Soybean meal	21.11	21.11	21.11
Wheat bran	20.00	20.00	20.00
L-lysine	0.19	0.19	0.19
L-threonine	0.10	0.10	0.10
L-tryptophan	0.06	0.06	0.06
L-methionine	0.12	0.20	0.28
Limestone	1.44	1.44	1.44
Dicalcium phosphate	1.51	1.51	1.51
Sodium chloride	0.40	0.40	0.40
Choline chloride	0.15	0.15	0.15
Vitamin premix ^1^	0.03	0.03	0.03
Mineral premix ^2^	0.50	0.50	0.50
Bentonite	0.64	0.56	0.48
Total	100.00	100.00	100.00
Calculated nutrient level (%)			
AME (MJ/kg)	10.87	10.87	10.87
CP	16.45	16.50	16.54
Calcium	0.95	0.95	0.95
non-phytic acid P	0.40	0.40	0.40
Dig lysine (Lys)	0.85	0.85	0.85
Dig methionine(Met)	0.32	0.40	0.48
Dig threonine (Thr)	0.60	0.60	0.60
Dig tryptophan (Trp)	0.22	0.22	0.22

AME = apparent metabolizable energy; CP = crude protein; Dig = digestibility. ^1^ Provided per kilogram of diet: vitamin A = 6875 IU; vitamin D_3_ = 1640 IU; vitamin E = 30.01 mg; thiamine = 1 mg; riboflavin = 3.9 mg; pyridoxine = 3.375 mg; vitamin B_12_ = 0.01 mg; calcium pantothenate = 8.85 mg; folate = 0.5 mg; biotin = 0.1 mg; niacin = 49.25 mg. ^2^ Provided per kilogram of diet: Cu (CuSO_4_∙5H_2_O) = 8 mg; Fe (FeSO_4_∙7H_2_O) = 80 mg; Zn (ZnSO_4_∙7H_2_O) = 90 mg; Mn (MnSO_4_∙H_2_O) = 70 mg; Se (NaSeO_3_) = 0.3 mg; I (KI) = 0.4 mg.

**Table 2:** Feather scoring criteria.

Characteristics	Grade	Score
Skin exposed, feather area less than 25%	0−	1
	0	2
	0+	3
Skin exposed, feather area between 50 and 70%	1−	4
	1	5
	1+	6
Feather area between 65 90%	2−	7
	2	8
	2+	9
Feather area greater than 90%, and the feather is wide, neat, and shiny	3−	10
	3	11
	3+	12

Adapted from Gustafson et al. (2007) [21].

**Table 3:** Effects of feeding regimens and dietary methionine on the growth performance of meat ducks from 29 to 49 days of age ^1^.

Item		29 d	49 d	29–49 d				49 d
Feeding Regimens	Met (%)	BW (kg)	BW (kg)	BWG (kg)	FCR (kg/kg)	FI (kg)	Met Intake (g)	BWU (%)
AD	0.32	2.19	3.54 ^a^	1.371 ^a^	4.14 ^b^	5.61 ^a^	17.96 ^e^	85.3%
AD	0.40	2.18	3.51 ^a^	1.324 ^a^	4.27 ^b^	5.60 ^a^	22.39 ^c^	79.0%
AD	0.48	2.18	3.47 ^a^	1.285 ^a^	4.22 ^b^	5.36 ^a^	25.71 ^a^	80.1%
FR	0.32	2.16	3.12 ^b^	0.973 ^b^	5.12 ^a^	4.82 ^b^	15.43 ^f^	88.6%
FR	0.40	2.20	3.14 ^b^	0.936 ^b^	5.37 ^a^	4.89 ^b^	19.56 ^d^	81.6%
FR	0.48	2.20	3.15 ^b^	0.957 ^b^	5.27 ^a^	4.96 ^b^	23.80 ^b^	87.2%
SEM		0.039	0.029	0.055	0.206	0.102	0.482	0.040
Main effects of feeding regimens								
AD		2.18	3.51 ^a^	1.327 ^a^	4.21 ^b^	5.52 ^a^	22.02 ^a^	81.48%
FR		2.18	3.14 ^b^	0.955 ^b^	5.25 ^a^	4.89 ^b^	19.59 ^b^	85.81%
Met level								
0.32%		2.17	3.34	1.172	4.63	5.22	16.69 ^c^	86.93%
0.40%		2.19	3.32	1.130	4.82	5.24	20.98 ^b^	80.33%
0.48%		2.19	3.31	1.121	4.75	5.16	24.75 ^a^	83.67%
*p* values								
ANOVA		0.977	<0.001	<0.001	<0.001	<0.001	<0.001	0.433
Feeding regimens		0.954	<0.001	<0.001	<0.001	<0.001	<0.001	0.186
Met level		0.856	0.645	0.617	0.663	0.683	<0.001	0.257
Feeding regimens × Met level		0.791	0.231	0.794	0.953	0.136	0.553	0.833

^1^ Means represent 10 pens per treatment of 60 ducks per pen. ^a–f^ Means within a column and under each main effect with no common superscripts differ at *p* < 0.05.

**Table 4:** Effects of feeding regimens and dietary Met on feather weights and back feather scores of meat ducks at 49 d of age ^1^.

Feeding Regimens	Met (%)	Feather Weight (g)	Feather Ratio %	Back Feather Score
AD	0.32	100.8 ^b^	2.97	5.95
	0.40	105.7 ^ab^	2.94	6.40
	0.48	111.5 ^a^	3.17	7.27
FR	0.32	104.7 ^ab^	3.27	6.32
	0.40	103.6 ^ab^	3.19	7.16
	0.48	98.70 ^b^	3.09	7.09
SEM		2.65	0.10	0.450
Main effects of feeding regimen				
AD		106.0	3.03	6.54
FR		102.3	3.18	6.85
Met level				
0.32%		102.8	3.12	6.14
0.40%		104.6	3.06	6.78
0.48%		105.1	3.13	7.18
*p* values				
ANOVA		0.026	0.130	0.223
Feeding regimens		0.095	0.057	0.400
Met level		0.646	0.755	0.075
Feeding regimens × Met level		0.009	0.106	0.585

^1^ Means represent 10 pens per treatment of 60 ducks per pen. ^a,b^ Means within a column and under each main effect with no common superscripts differ at *p* < 0.05.

**Table 5:** Effects of feeding regimens and dietary Met on feather length in meat ducks at 49 days of age ^1^.

Feeding Regimens	Met (%)	Head	Neck	Back	Chest	Belly	Wing
AD	0.32	19.6 ^b^	31.1 ^a^	53.35	53.5 ^c^	47.9	160.7 ^ab^
	0.40	22.7 ^ab^	24.5 ^b^	60.65	56.0 ^bc^	50.4	173.6 ^a^
	0.48	24.0 ^a^	27.7 ^ab^	58.65	57.5 ^ab^	50.6	165.4 ^ab^
FR	0.32	20.1 ^b^	27.9 ^ab^	57.22	57.2 ^ab^	51.0	174.8 ^a^
	0.40	24.7 ^a^	26.6 ^b^	59.8	57.8 ^ab^	50.6	156.0 ^b^
	0.48	23.5 ^a^	24.8 ^b^	59.94	60.2 ^a^	50.4	164.4 ^ab^
SEM		1.08	1.33	2.110	1.22	0.79	4.61
Main effects of feeding regimens							
AD		22.1	27.8	57.6	55.6 ^b^	49.6	166.6
FR		22.8	26.4	59.0	58.4 ^a^	50.7	165.0
Met level							
0.32%		19.8 ^b^	29.5 ^a^	55.3	55.3 ^b^	49.5	167.7
0.40%		23.7 ^a^	25.6 ^b^	60.23	56.9 ^ab^	50.5	164.8
0.48%		23.8 ^a^	26.3 ^b^	59.3	58.8 ^a^	50.5	164.9
*p* values							
ANOVA		0.005	0.012	0.170	0.011	0.088	0.041
Feeding regimens		0.436	0.232	0.408	0.008	0.116	0.690
Met level		0.001	0.011	0.054	0.022	0.314	0.768
Feeding regimens × Met level		0.507	0.093	0.539	0.743	0.082	0.005

^1^ Means represent 10 pens per treatment of 60 ducks per pen. ^a–c^ Means within a column and under each main effect with no common superscripts differ at *p* < 0.05.

## Data Availability

The dataset is available upon request from the authors.
